# Monthly Summary

**Published:** 1893-07

**Authors:** 


### Monthly Summary.

A DIFFICULT CASE TO CONTROL.—By Carl E. Klotz, St. Catharines, Ont.—A lady of about forty-five years of age has, since her childhood, been inclined to nausea and vomiting upon the slightest provocation. She came to me for an entire denture. Her teeth, or rather the remains of them, were extracted under the influence of chloroform.

About three weeks after this, the patient came by appointment to have her mouth examined preparatory to having the impressions taken; but as soon as either the fingers or the mouth-glass entered her mouth, the trouble began. Up to this date I knew nothing of her complaints, as she showed no signs of it, nor said anything about it when she first consulted me. She told me now, and also said she found it very remarkable that she could that day tolerate the mouth-glass, when I was examining her mouth, without producing the usual disagreeable effect.

A gargle of bromide of potassa was given her, and the impression and articulation could be taken without difficulty. A trial plate was made of impression compound, and the teeth set up on it, ready to be tried in her mouth at the next sitting; this was attempted, but here the circus began.

Having taken the precaution to use the bromide gargle before the plates were inserted, I expected to get along as nicely as I did with the impressions, but not so. The bromide had no effect whatever, and, after trying different means, I finally succeeded with cocaine, not by gargling, but by painting over the whole roof of the mouth and over the alveolus with a pellet of cotton dipped into a twenty per cent. solution of cocaine. The plates could now be fitted. After having finished them I inserted them at the next sitting, having used the cocaine as previously, without any trouble whatever.

The upper denture fitted so well that force had to be used to remove it, but the old trouble soon came back, for the patient, after wearing the dentures several weeks, could not keep the upper in her mouth. In fact, it would not stay up. She came to the office, and I could do no better than paint the same as before. All appeared well. The plate remained in its place and there was no inclination to nausea, but I had to repeat this frequently for several weeks, her own treatment at home, as I instructed her, having very little effect. This was getting monotonous, although I was getting to be quite an artist at painting; but it effected no cure. The idea struck me to try a spray, which was done, using the following with an atomizer :

| | | |
|---|---|---|
| Cocaine, | - - | grs. viii. |
| Syr. Tolutanus, | - - | ℥ ss. |
| Spts. Vin. Roe, | - | ʒ iii. |
| Aqua, | - - - | ℥ ii. |

and this was attended with happy results.

I gave her instructions to spray her mouth every morning and evening and, if need be, during the day for two weeks, then gradually diminish to once a day for another week, after that only when sensation of nausea came on.

It is now several months since she used the spray. Since then she has been very comfortable. Although the time is rather short to be assured of a cure, yet, taking into consideration that she has been troubled with this malady since her childhood, and has scarcely been a day without an attack, it appears very favorable for a permanent cure.—*Dominion Dental Journal.*

---

A MISPLACED CANINE.—Dr. Walker Downie has communicated to the Medico-Chirurgical Society of Glasgow the details of a case of chronic suppuration in the antrum of a little girl, aged six years. Regular poulticing under the direction of a "bone-setter" had been carried on for six months. The right eye was partially closed, and the nose pushed over to the left. There was a foul-smelling, purulent discharge from the right nostril, and extensive ulceration of mucous membrane of the mouth, immediately above the canine tooth. A probe demonstrated necrosed bone over a considerable area, and, under chloroform, the right lateral and canine (both carious) were removed, and afterwards the anterior wall of the antrum. Much pus escaped, and when the cavity had been washed out, a hard body was felt at the upper and anterior portion of the cavity, firmly attached to the wall, but projecting from it. On removal, it was recognized as a permanent canine tooth, with the crown large and fully developed, and the fang in a forward condition. We agree with the remark, that it is difficult to account for the position of the tooth, but the unusual development would prepare one for eccentric behavior on the part of such a precocious canine, which, normally, should have made its appearance some six years later.—*British Journal.*

---

CAMPHO-PHENIQUE is spoken of very highly by Professor O. N. Bedell, of St. Louis. He says: I have used it with much benefit in after-pain of teeth extraction, especially of ulcerated teeth; also in pain and tenderness of teeth from poisoning, salivation, necrosis of alveolar, and in many cases of inflammation, pulpitis, pericementitis, alveolar abscess, stomatitis, etc. No student or practitioner of dentistry can well say his list of remedies is complete without campho-phenique being represented.—Items.

FERMENTATION.—Dr. Hoff in the *Dental Register* treats this subject as follows: It is the means by which existing organizations are broken down into molecular conditions, so that growth and development may take place and even life itself be perpetuated. For instance, food taken into the alimentary canal meets first with a substance in the saliva called ptyalin, which changes its starch into a substance that can be appropriated by those absorbent structures whose business it is to secure the pabulum for the proper nutrition of the tissue of the body. Gastric juice and the pancreatic fluid each contain a peculiar ferment which selects its peculiar kind of food and breaks it up into its elements, so that the absorbents may appropriate them to nourish growing tissue cells. We have an excellent illustration in the rotting of grain where a peculiar substance called diastase, has the property of changing the nature of the starch contained in the grain to an entirely different substance, so that the plant that is to grow from the seed may appropriate it for nourishment.

This class of ferments are called organic, because they are the product of an organized body, but they possess no organization, such as we find in another important class of ferments which are organized.

The organic ferments are chemical but not vital substances, and in the economy of nature they are useful agencies in procuring food and nourishment for organized living bodies, but are not largely concerned in the destruction of living tissue in the production of disease.

The organized ferments have a distinct and definite organization. They, under favorable conditions, exhibit all the phenomena of life. They are capable of locomotion, grow and reproduce their kind. They exhibit many phenomena of animal life, yet microscopic investigation has determined their place as belonging to the vegetable kingdom. They are essentially seeds of plants—a body of protoplasm and a cellular wall or covering of a ligneous nature. Some are capable of destroying life in tissue and creating disease, and are called pathogenic; others are harmless or non pathogenic. Some need air or oxygen to support life, erobic; others live without air and are anerobic. It is therefore folly to think of all these germs as destructive. We could not live without them. Many of those we speak of as destructive destroy substances much more noxious than themselves.

---

ABSCESS OF THE ANTRUM OF HIGHMORE.—J. M. Hunt, in the *Liverpool Medico-Chirurgical Journal*, publishes some important points for consideration in an interesting paper on this disease.

In the author's experience the so-called classical symptoms, (1) distention of the antrum, (2) swelling of the cheek, (3) infraorbital pain, (4) escape of pus on the sound side, are, as a rule, conspicuous by their absence. The one constant and all-important symptom is the presence of a purulent nasal discharge coming from the concavity of the middle turbinate, and escaping either by the anterior or posterior nares. Pain is, as a rule, present, and is generally intermittent in nature and supraorbital in position. As regards the intranasal condition, diffuse hypertrophy of the middle turbinate, polypoid degeneration or even true polypi in the middle meatus, hypertrophy of the mucous membrane in the neighborhood of the hiatus semi-lunaris, bare bone in the middle turbinate or at the ostium maxillary are to be found in most cases. The author regards it as justifiable to open the antrum in any patient with unilateral purulent discharge coming from the concavity of the middle turbinate in its anterior half, and constantly or at times ill smelling, if there be no obvious cause for the discharge inside the nose itself, such as a foreign body or specific ulceration. He does not regard the trans-illumination of the antral cavities by means of Voltolini's electric lamp placed in the mouth as of much practical importance, although in a doubtful case it may at times be useful. Exploratory puncture carried out through the middle or inferior meatus with a Pravaz syringe or a Lichtwitz trochar and canula is a more reliable method. For purposes of treatment, the best plan is to open the antrum from the alveolar process, either through the socket of a tooth which has been removed or from the alveolar border.

PERIOD OF INFECTIOUSNESS.—The period of infectiousness of contagious diseases, according to the State Health Board of Pennsylvania, is:

Small-Pox—Six weeks from the commencement of the disease, if every scab has fallen off.

Chicken-Pox.—Three weeks from the commencement of the disease, if every scab has fallen off.

Scarlet-Fever—Six weeks from the commencement of the disease, if the peeling has ceased and there is no sore nose.

Diphtheria—Six weeks from the commencement of the disease, if sore throat and other signs of the disease have disappeared.

Measles—Three weeks from the commencement of the disease, if all rash and the cough has ceased.

Mumps—Three weeks from the commencement of the disease if all swelling has subsided.

Typhus—Four weeks from the commencement of the disease, if strength is re-established.

Typhoid—Six weeks from the commencement of the disease, if strength is re-established.

Whooping Cough--Six weeks from the commencement of the disease, if all cough has ceased.

Under judicious treatment the period of infectiousness may be considerably shortened, but *no child suffering as above should be admitted to any school after a shorter period of absence, and then should be provided with a medical certificate*, that he or she is not liable to communicate the disease.

Length of Quarantine.—Teachers or children *who have been exposed* to infection from any of the following diseases may safely be re-admitted to the school, if they remain in good health (and have taken proper means for disinfection) after the following periods of quarantine:

Diphtheria, 12 days; scarlet fever, 14 days; small-pox, 18 days; measles, 18 days; chicken-pox, 18 days; mumps, 24 days; whooping-cough, 21 days.

Adults may be re-admitted immediately, if they disinfect their clothes and persons.—*Md. Med. Journal.*

EXEMPTION OF DENTISTS FROM JURY DUTY.—Wyoming seems to have been in advance in this important movement. We copy the following from a private letter received from Dr. R. J. Gardinier, of Laramie, Wyoming:

" 'I see by one of the late dental journals that the point is being agitated of exempting dentists from jury duty (and rightly so). Wyoming is somewhat ahead in this line, at least. In the year 1885, when our Territorial laws were in the hands of the committee to be compiled, I presented the claims of dentists for exemption so strongly and satisfactorily, that my wish was granted; so that dentists were included as exempt from jury duty.' "

The above, clipped from an exchange, is news to me, as I have been under the impression that dentists, in all the States alike, were exempt from jury duty. In the discussions in the Mississippi Society, a few weeks ago, I notice that some of the members complain of having to do jury duty. Georgia has never required dentists to do jury duty, and I feel sure that the profession, in all the States, could be exempted if the proper steps were taken. The various State societies should take the matter up for consideration, and have the law so changed.

Physicians are exempted because their services are liable to be required at an unexpected moment for the preservation of life or relief of suffering. Patients of dentists have the same right to require the services of a dentist when needed, and only a little effort would be required to have the laws so changed.—*Southern Dental Journal.*

---

DR. CHAS. B ATKINSON says that a twenty-five per cent. pyrozone, ethereal solution, is probably the best bleacher for teeth that has ever been offered. Its effects is exceedingly prompt and the results are permanent. The process is not attended with pain unless the gums be touched, when a severe pricking sensation is produced, and a coagulum seems to form in most cases; but this will return to a normal condition if not abraded. He also recommends it in treating abscess pockets and suppurating pyorrhœa alveolaris.—*Items.*

TREATMENT OF THE EAR.—Poulticing the ear may seem to be a simple operation, but there is nevertheless a right and wrong way of doing it, and it appears that the wrong way is the one usually adopted. Dr. Buck says that while heat is one of the best remedies in painful inflammations of the middle ear, and the poultice is one of the best methods of applying heat, as usually put on the poultice has little effect. What should be done, he says, is first to fill the external auditory canal with lukewarm water, the head resting on the unaffected side upon the pillow. Then a large flaxseed poultice is applied over the ear as hot as it can be borne. The column of water is thus kept warm and acts as a conductor of heat between the poultice and the inflamed surface.—*N. Y. Med. Times.*

---

A HINT.—By T. L. HALLETT, M. D. S.—A pivot tooth which, for durability, I consider second to none, is made by using silver for pivot and backing, and soldering with silver solder, and setting with Weston's cement mixed thin and amalgam soft, about equal parts, and finishing part exposed to fluids of the mouth with amalgam only. The amalgam unites with silver only sufficient to make it all like one piece of metal, while the cement setting quickly avoids any danger of displacement of the tooth. With that setting, gold, of course, must be strictly avoided as backing or pivot.—*Dominion Dental Journal.*

---

COME TO OUR ARMS!—"Dental colleges are like individuals, careful or slovenly, just as they happen to be manned or officered. A great improvement could be made in various portions of the country if colleges were endowed."

Many colleges stand with open arms, ready to receive endowments to almost any number of hundred thousand; and will not be particular who the donors are, either; and we guarantee that all who have donations to make can be accommodated.—*Dental Review.*

Antiseptic Properties of Tobacco.—Professor Tassinarini, in Rome, calls attention to the fact that as far back as in the seventeenth and eighteenth centuries, the use of tobacco was recommended by many physicians as a prophylactic in times of epidemics. In 1842 Professor Ruef, in Strasburg, it is said, stated that all the workingmen employed in the Royal Tobacco Manufactory had remained exempt from contagious diseases. Recholier made the same observations in 1883, and Walter Cock, of Texas, in 1889, even recommended tobacco-smoking as a preventive against tuberculosis. Dr. Vassili, of Naples, in 1888, experimented on a balloon in which a layer of gelatine was sprinkled with comma bacilli. As soon as the smoke from one to four cigars, according to the intensity of the tobacco, was introduced into the balloon, the micro-organisms of the gelatine were completely killed. These various experiments were imitated by Tassinarini and he found that by tobacco smoke the comma bacilli, the bacilli of pest and of pneumonia are destroyed, or at least arrested in their development. In these experiments it was observed that the bacilli of cholera and of pneumonia were destroyed within a few minutes, while the bacillus of pest offered more resistance, and while the bacillus of typhus was scarcely interfered with. Tassinarini also affirms that smoking delays caries of the teeth, for which reason women, it is alleged, are more afflicted with diseased teeth than men. All these views, of course, should not be accepted as infallible without further demonstration.—*Internationaler Pharmac. General Anzeiger.*

---

Soldering Aluminum.—The soldering of aluminum which has long been a difficult problem, has been recently solved. By sprinkling the surface to be soldered with chloride of silver, and melting down, the soldering is effected simply and satisfactorily.—*Ohio Journal.*

